# Cellular Senescence and Regulated Cell Death of Tubular Epithelial Cells in Diabetic Kidney Disease

**DOI:** 10.3389/fendo.2022.924299

**Published:** 2022-06-28

**Authors:** Shuang Shen, Chuanyuan Ji, Kaifeng Wei

**Affiliations:** College of Traditional Chinese Medicine·College of Integrated Traditional Chinese and Western Medicine, Nanjing University of Chinese Medicine, Nanjing, China

**Keywords:** regulated cell death, diabetic tubulopathy, cellular senescence, diabetic kidney disease (DKD), regulated cell death (RCD)

## Abstract

Cellular senescence is frequently evident at etiologic sites of chronic diseases and involves essentially irreversible arrest of cell proliferation, increased protein production, resistance to apoptosis, and altered metabolic activity. Regulated cell death plays a vital role in shaping fully functional organs during the developmental process, coordinating adaptive or non-adaptive responses, and coping with long-term harmful intracellular or extracellular homeostasis disturbances. In recent years, the concept of ‘diabetic tubulopathy’ has emerged. tubular epithelial cells are particularly susceptible to the derangements of diabetic state because of the virtue of the high energy requirements and reliance on aerobic metabolism render. Hyperglycemia, oxidative stress, persistent chronic inflammation, glucose toxicity, advanced glycation end-products (AGEs) accumulation, lipid metabolism disorders, and lipotoxicity contribute to the cellular senescence and different patterns of regulated cell death (apoptosis, autophagic cell death, necroptosis, pyroptosis, and ferroptosis) in tubular epithelial cells. We now explore the ‘tubulocentric’ view of diabetic kidney disease(DKD). And we summarize recent discoveries regarding the development and regulatory mechanisms of cellular senescence, apoptosis, autophagic cell death, necroptosis, pyroptosis, and ferroptosis in the pathogenesis of DKD. These findings provide new perspectives on the mechanisms of DKD and are useful for designing novel therapeutic approaches for the treatment of DKD.

## Introduction

In the past 30 years, diabetic kidney disease (DKD) has been considered as the leading reason for end-stage renal disease (ESRD) (1), with an increasing trend worldwide. Although significant progress has been recently achieved in the pathogenesis and clinical therapy for DKD, there is still an unmet need for a better control of DKD progression. DKD is clinically featured by albuminuria development and a decrease in glomerular filtration rate (GFR), accompanied by glomerulosclerosis, tubulointerstitial fibrosis (TIF), and atrophy (2). Moreover, DKD is a typically progressive microvascular complication arising from diabetes (3). The current research predominately focuses on the glomerulus, as glomerular change is critical for DKD. However, it is neither a primary determinant of renal prognosis in diabetes nor a significant event in the progression of DKD. Renal tubules and tubulointerstitial account for 90% of the mass of renal parenchyma, which indicates that they are critical for the development of DKD.

Recently, the role of cellular senescence in DKD has attracted extensive attention. Cellular senescence in DKD involves various mechanisms, including telomere shortening, DNA damage, epigenetic modifications, and mitophagy deficit (4). Cell death can be divided into accidental cell death (ACD) and regulated cell death (RCD) based on functional differences. RCD includes recognition, triggering, execution, and other effector molecules and produces signal cascade reaction, which has unique biochemical characteristics, morphological characteristics, and immunological consequences. RCD plays a vital role in shaping fully functional organs during the developmental process, coordinating adaptive or non-adaptive responses, and coping with long-term harmful intracellular or extracellular homeostasis disturbances. When the cellular stress responses are unable to respond to these disturbances, cell death is a side effect of attempting to restore balance, regardless of the outcome (5).

The most typical RCD pattern is apoptosis. Many forms of non-apoptotic RCD have been described in recent years. These symptoms include autophagic cell death, pyroptosis, necroptosis, ferroptosis, parthanatos, alkaliptosis, netosis, entosis, oxeiptosis, and so on (5, 6). It should be noted that a variety of types of cell death occur in DKD, including apoptosis, autophagic cell death, necroptosis, pyroptosis, and ferroptosis. In this review, we reconsider the current “glomerulocentric” paradigm, tune the focus to the proximal tubule, summarize various forms of evidence of cellular senescence and the RCD of renal tubular epithelial cells, describe its underlying mechanism, emphasize new insights, and provide perspective on the prevention and treatment of DKD by targeting cell death.

## Renal Tubular Injury is an Essential Link in the Pathogenesis of DKD

Glomerular structural changes, such as mesangial expansion, reduction in the capillary surface, and podocyte loss, are undoubtedly the main features distinguishing DKD from other types of glomerulonephritis (1). However, the renal tubules of patients with diabetes undergo a series of structural changes, such as renal tubule atrophy, interstitial fibrosis, and peritubular capillary rarefaction, each of which is closely related to the decline of renal function (7). Especially, a close correlation between the extent of tubulo-interstitial injury and long-term renal function has been demonstrated (8). Firstly, the high demand for oxygen in the kidneys is primarily caused by the proximal tubules’ huge reabsorption function. The reabsorptive process is an active transport, which in turn makes it particularly vulnerable to hypoxia. High energy demand and dependence on aerobic metabolism make renal tubular epithelial cells particularly sensitive to disorders in the diabetic state. hyperglycemia can independently lead to acute renal tubular necrosis, tubular cell apoptosis, epithelial-mesenchymal transition, and ECM deposition. In the early stage of glucose metabolism disorder, proximal tubule hypertrophy due to increased glucose reabsorption is associated with reactive oxygen species production, oxidative damage, and TGF-β production. These effects lead to proximal tubule cell G1 cell cycle arrest and senescence phenotype, thereby promoting interstitial inflammation and fibrosis (9, 10). Secondly, tubulopathy is not secondary to the glomerulus, while its early and initial characteristic changes (11). Hypertrophy of renal tubules can be observed in diabetic kidneys several days after hyperglycemia onset. Besides diabetic glomerular alternations, even in patients with normal albuminuria, the increase in the width of the tubular basement membrane (TBM) can be detected as one of the early-stage structural abnormalities in the diabetic kidney (12). However, some studies have shown that up to 51% of patients with diabetes may have kidney damage without prior proteinuria. Russo et al. proved that the function of proximal tubule handling of albumin in hyperglycemia state is abnormal, resulting in albumin-derived peptinuria, excreted in the nephrotic range, which is not related to the change of glomerular capillary wall permeability (13). We recognize that many patients with diabetes and low GFR do not have significant albuminuria and that the decrease of GFR often precedes the occurrence of microalbuminuria. Thirdly, It was found that about 7% of patients showed non-functioning glomeruli atrophy happening at the critical junction between Bowman’s capsule and the proximal tubule (14). In the absence of obvious albuminuria, 26% of the patients still show abnormal glomerulotubular junction, which was negatively correlated with creatinine clearance (r = 20.70, P < 0.05) (14). In groundbreaking research in 2013, Hasegawa et al. provided evidence of retrograde trafficking between the proximal renal tubules and the glomerulus (15), indicating that nicotinamide mononucleotide (NMN) was released by proximal renal tubular epithelial cells diffused back to the glomerulus, resulting in podocyte foot process disappearance and proteinuria (16). Other studies have shown that proximal tubular injury leads to podocyte damage and more extensive glomerular injury. Fourthly, recent studies have found that renal tubular albuminuria is a predictor of DKD progression (17). In conclusion, tubulopathy is the prime and important factor that influences the progression of DKD and it is becoming more and more challenging to ignore renal tubular damage.

## Cellular Senescence

Previous research has demonstrated that DKD is highly correlated with the accelerated senescence of renal tubular epithelial cells, mesangial cells, podocytes, and endothelial cells (18, 19). It is worth noting that hyperglycemia can also induce macrophages to secrete senescence-associated secretory phenotype components (SASP), and promote the development of low-grade inflammation, directly inducing Cellular senescence of mesangial (20) and renal tubular epithelial cells (21). The accumulation of damaged mitochondria during DKD may be responsible for the premature senescence of renal tubular epithelial cells. Under the condition of DKD disease, in addition to hyperglycemia, oxidative stress, persistent chronic inflammation, glucose toxicity, advanced glycation end-products (AGEs) accumulation, lipid metabolism disorders, and lipotoxicity contribute to the formation of a growth microenvironment that promotes Cellular senescence. Telomeres, as exceptionally conserved tandem nucleotide repeats, can protect the ends of chromosomes to retain the genome’s integrity and take place age-related gradual attrition (22). Shorten and inactive telomeres are thought to be DNA double-strand fractures that initiate Cellular senescence. In patients with diabetes, chromosomal telomere loss is related to renal Cellular senescence, proteinuria, and DKD progression (23, 24). Oxidative stress is considered to be one of the most critical reasons for telomere shortening, indicating an imbalance between antioxidants and reactive oxygen species (ROS) (25, 26). DNA damage is seen as the primary cause of cellular senescence. Under diabetic conditions, the hyperglycemia-mediated production of ROS and accumulation of AGEs would lead to DNA damage, which in turn causes premature senescence of cellular glomeruli and renal tubular epithelial cells (27).

Epigenetic modifications, such as cytosine DNA methylation, histone post-translational modifications (PTMs), and noncoding RNAs, coordinate the relationship between genes and the intracellular environment, which only affect genes expression and function instead of influencing the DNA sequence (28). Nutritional status, lifestyle, and environmental factors strongly affect the occurrence and development of diabetes and DKD through epigenetic alternations (29). The experimental evidence supports that DNA methylation is involved in glomerular and proximal tubular epithelial cells’ normal function, such as filtration, glucose, and solute handling (30). In the *db/db* mouse model of DKD, the transcriptional inhibition of transcription factor KLF4 increases DNA methylation (31). A 2017 study showed that the proximal tubular cell cultured in a high glucose environment and the kidneys harvested from mice pretreated with streptozotocin (STZ) showed decreased DNA methylation of MIOX, correlated with the enhanced expression of the encoded protein inositol oxygenase (32). PTM-regulated gene expression of nucleosome histones in chromatin is an integral part of epigenetic regulation (33). The primary histone PTMs include lysine acetylation, methylation, ubiquitylation, serine/threonine phosphorylation, and arginine methylation (34). Several *db/db* mouse model studies of type 2 diabetes mellitus (T2DM) found that different chromatin-based histone PTMs were observed in the promoters of PAI1 and AGER (encoding age-specific receptor RAGE) and enhanced the RNA polymerase II recruitment, suggesting that activating and inhibitory epigenetic markers jointly facilitate chromatin unwinding and improve the recruitment of transcription factors to the genes associated with DKD. In addition, the increase of renal inflammation caused by macrophage infiltration is one of the pathogenesis of DKD. In a high glucose circumstance, histone modification mediates the activities of NF-κB and other inflammatory cytokines in vascular cells and monocytes (35, 36). Epigenome ncRNAs, such as small ncRNAs (sncRNAs), miRNAs, and long ncRNAs, show critical regulatory functions. miR-192 is the first miRNA demonstrated to play a functional role in DKD, promoting the expression of extracellular matrix (ECM) and collagen by targeting key inhibitors and enhancing the fibrogenic effect of TGF-β (37). lncRNA PVT1 participates in the pathogenesis of DKD (38). Under high glucose conditions, miRNAs1204-1208 located at the PVT1 site were also found to upregulate the expression of ECM-related gene in human mesangial cells (39). The underlying mechanisms of glucose metabolism memory and cell death in diabetes may be related to the long-term persistence of epigenetic modifications and ncRNAs. For instance, lncRNA MALAT1(metastasis associated in lung denocarcinoma transcript 1) inhibits the expression of miR-23c, thereby regulating the pyroptosis of renal tubular epithelial cells in a DKD model. Epigenetic and epigenomic alternations have the potential to develop new biomarkers and treatments for diabetes or DKD. It has been evident that many renal tubular epithelial cells (>50%) display mitochondrial fragmentation and autophagy loss under diabetic conditions (40), accompanied by apparent upregulated mtROS levels in the renal cortex and massive production of ROS (41). In addition, functional loss of Klotho protein, activation of the Wnt/β-catenin signaling pathway, and accumulation of inflammatory cytokines and uremic toxins may also contribute to the network of DKD Cellular senescence.

## Apoptosis

Apoptosis is an RCD process characterized by volume reduction, cell surface blistering, chromatin condensation, DNA nucleosome cleavage, and apoptotic body shape. Two apoptosis pathways have been found in the mammalian system, including the death factor pathway and extrinsic death pathway. Caspase-3, at the end of the caspase cascade, can be stimulated by endogenous and exogenous death pathways, suggesting that caspase-3 substrates mediate the general features of apoptosis (42). The renal tubular epithelial cell apoptosis is the primary characteristic of DKD. The *in vitro* and *in vivo* experiments performed by Guo et al. found that the apoptosis of renal tubular epithelial cells was elevated significantly under high glucose conditions. Furthermore, Calcitriol treatment attenuates tubular epithelial cell apoptosis in DKD by targeting the Vitamin D receptor. Hyperglycemia can cause the generation of free radicals and oxidative stress in renal tubular cells (43). On the other hand, ROS contributes to various biological processes, such as proliferation, extracellular matrix deposition, and apoptosis. The activation of apoptosis signal-regulated kinase-1 (ASK-1), an upstream kinase of the p38 mitogen-activated protein kinase (MAPK) pathway, is due to inflammation and oxidative stress, which would result in renal fibrosis and promote DKD development (44).

## Autophagic Cell Death

Autophagic cell death is a unique RCD pathway, which does not require caspase and does not have typical morphological features of apoptosis, necroptosis, or pyroptosis. The accumulation of many vacuoles in the cytoplasm of the autophagy dead cells is considered one of autophagy death features (45). It is still debated that the existence of an autophagic cell death pathway as a bona fide active mechanism for cellular killing (6). Moreover, it is catabolic and homeostatic progress in lysosomes to degrade damaged organelles, protein aggregates, and recycled materials, and it is the last line of defense to maintain cellular viability (46). Once the nutrient deficiency persists, it progresses to autophagic cell death which is irreversible. Autophagy-related gene (ATGs) is a specific gene family responsible for The initiation, formation, and maturation of autophagosomes are controlled by one particular ATG family. And autophagy fuses with lysosomes and hydrolyzes/degrades encapsulated substances through autophagy (47). This comprehensive process is mainly modulated by two widely expressed proteins, the mammalian target of rapamycin (mTOR) and AMP-activated protein kinase (AMPK). mTOR complex 1 (mTORC1) is the primary receptor of intracellular amino acids, facilitating protein synthesis (anabolism) and inhibiting autophagy. Autophagy can be significantly stimulated by starvation, energy exhaustion, and hypoxemia. Autophagy is thought to keep cells alive by circulating amino acids and the necessary cellular metabolites (48). Impaired autophagy in patients with diabetes may be caused by high glucose, leading to the accumulation of unfolded proteins and dysfunctional organelles. Therefore, suppressing the clearance of damaged cell contents, such as AGEs, can result in renal cell damage and fibrosis regulated by TGF-β (49). It is widely evident that fully functional autophagy is essential to delay the incidence of DKD. The expression of SGLT2 mainly occurs in the brush borders of the proximal tubule segments of S1, which is enhanced by 40-80% with the increase of persistent glycosuria (50). The increased expression of SGLT2 can maximize glucose reabsorption and improve Na^+^-K^+^-ATPase activity and oxygen consumption, resulting in hypoxia and tubular injury. Therefore, inhibiting SGLT2 can enhance the oxygenation of proximal tubules. Recent findings suggest that SGLT2i enhances ketolysis and autophagy while inhibiting mTORC1 expression (51). Proximal tubules are the main target of putative SGLT2i effects, but a study reported an upregulation of podocyte SGLT2 expression in a murine model of protein-overloaded proteinuria induced by bovine serum albumin (BSA), and treatment with SGLT2i dapagliflozin significantly improved albuminuria *via* restoration of cytoskeletal remodeling in the podocytes (52). It has been reported that, in DKD, the activation of Smad3 triggers autophagy dysfunction and promotes DKD (53). Smad3 may bind and inhibit the expression of TFEB at the transcriptional level, thereby suppressing the biogenesis of lysosomes and damaging the clearance of damaged lysosomes, which results in the depletion of lysosomes in the TECs of renal tubular epithelial cells during diabetes. Another study showed that, in PTECs cultured with high glucose, the levels of Beclin1 and LC3-II were increased, while the p62 level was reduced, which indicates that autophagy performance is upregulated in diabetes. In addition, autophagy deficiency due to autophagy inhibitors (e.g., chloroquine and 3-methyladenine) and ATG5 siRNA transfection aggravates lipid accumulation and EMT (54). Therefore, autophagy can be targeted to develop therapeutic interventions for the prevention or relief of DKD.

## Necroptosis

Necroptosis is a cellular response to environmental stress, which can be attributed to chemical and mechanical influences, inflammation, or infection. There is growing evidence from genetic and pharmacological studies proving that necroptosis is critical for the etiology and progression of many human disorders, including cell injury induced by ischemia-reperfusion, inflammation caused by a viral infection, inflammatory bowel disease, and neurodegenerative diseases (55, 56). Necroptosis is featured by swelling, loss of plasma membrane permeability, and membrane rupture (57). It can also release damage-associated molecular patterns (DAMPs), such as HMGB1, S100 protein, ATP, IL-33, IL-1α, HSP70, double-stranded DNA (dsDNA), and mtDNA, and promote the production of cytokines (e.g., IL-6 and IL-1β) associated with inflammation (58). Therefore, necroptosis is different from apoptosis in morphological and biological consequences. Receptor-interacting protein kinase 3 (RIPK3) and the related substrate MLKL are seen as critical regulators of necrosis. Moreover, RIPK1 and RIPK3 independently influence inflammation without affecting cell death (59). Earlier studies have shown that RIPK3-related necroptosis is the primary form of renal tubular epithelial cell death in rats with chronic renal injury (60). The hyperglycemia/AGEs-activated intrarenal RAAS system can increase the levels of renin and angiotensin in renal cells (61, 62). On the other hand, it has been reported that the cytotoxicity of AngII can cause renal tubular epithelial cell necroptosis *in vitro* and *in vivo* and then elevate the levels of the regulatory factors Fas and FasL that are involved in cell death induced by angiotensin-converting enzyme. In addition, pharmacological inhibition using AngII and FasL inhibitors or RIPK1/3 and MLKL blockers suppressed excessive death of renal tubular epithelial cells mediated by AngII *in vitro* and *in vivo* (60). Another study showed that RIPK3 deficiency in UUO models avoided fibrosis kidneys, which is a common pathway of DKD.

## Pyroptosis

Persistent inflammation of circulation and kidney tissue is considered to be a crucial pathophysiological basis of DKD. In patients with diabetes, the inflammatory transcriptional signal NF-κB can be activated simultaneously by high glucose, AGEs, and oxidative stress (63). Pyroptosis is an inflammatory type of RCD driven by inflammatory caspase 1, 4, 5, and 11. Pyroptosis is mainly an innate immune response to intracellular pathogens, which is performed by caspase-dependent GSDMD cleavage. In the process of pyroptosis, the cleavage fragment of GSDMD at the amino-terminal permeates the plasma membrane and forms membrane pores, promoting the release of cytokines (e.g., IL-1β and IL-18) associated with inflammation. The increased circulation levels and pro-inflammatory cytokines IL-1β and IL-18 can cause persistent inflammatory effects, resulting in kidney damage in diabetes patients. Similarly, the experimental results of Sassy-Prigent et al. showed that the expression of IL-1β in renal tissue is upregulated in the DKD animal model (64).

In a typical caspase-1-mediated pyroptosis model, the activation of intracellular polyprotein signaling complexes (inflammasomes) is attributed to the recognition of inflammatory ligands. As a family of NOD-like receptors, the role of inflammatory bodies containing pyrrole domain 3 (NLRP3) in DKD has attracted much attention. NLRP3 inflammasome comprises nucleotide-binding and oligomerization domain-like receptor family NLRP3, and apoptosis-related spot-like proteins, such as caspase recruitment domain (ASC), and caspase-1 or caspase-5, in which NLRP3 is the essential protein. NLRP3 inflammasome is a potent inflammatory mediator that can activate caspase-1, IL-1β, IL-18, and other cytokines. Hyperglycemia, hyperlipidemia, and hyperuricemia can activate pyroptosis caused by the NLRP3 inflammasome. Two signaling pathways are required to activate NLRP3 inflammasome: a. the first pathway induces the transcription of NF-κB-dependent NLRP3 and cytokines through Toll-like receptors (TLR) and cytokine receptors as primers for NLRP3 activation; b. the second pathway mediates/activates NLRP3 inflammasome through unclear mechanisms, promoting the initiation and progression of DKD mediated by the models of the potassium channel, lysosomal damage, as well as active oxygen cluster (65). In renal patients, NLRP3 inflammasome corpuscle activation exists not only in immune cells (primarily located in macrophages and dendritic cells) but also in some inherent renal cells, such as renal tubular epithelial cells. IL-1β and IL-18 are produced by mouse tubular epithelial cells. It has been reported that these types of cells consist of all the required components to activate the inflammasome (66). On the other hand, mouse glomerular endothelial cells, mesangial cells, and podocytes are not responsible for IL-1β production or caspase-1 activation. NLRP3 is considered to be positively associated with renal tubular epithelial-mesenchymal transformation. The enhancement of TGF-β signal promotes renal tubular epithelial-mesenchymal transformation (67). It was found by Wang et al. that, in STZ-induced DKD with hyperuricemia and hyperlipidemia, ASC and caspase-1 were overexpressed, and the levels of IL-1 β and IL-18 were elevated (68). It was reported by Kim et al. that uric acid-mediated inflammatory activation of NLRP3 improves chemokine signal transduction in the proximal tubules of renal cells, thus facilitating DKD development associated with the regulation of NLRP3 inflammatory bodies. Additional studies have shown that mtROS is the primary activator of the NLRP3 inflammasome (69).

## Ferroptosis

It has been demonstrated that the accumulation of ROS and hemochromatosis are critical determinants of DKD in diabetes patients (70, 71). Excessive iron in cells impairs cell function through the production of ROS, and eventually leads to cell death (72). Ferroptosis is a pattern of RCD featured by the accumulation of iron-dependent lipid hydrogen peroxide to fatal levels, which has been considered a significant cause of cell death related to a range of indications, such as diabetes, cancer, neurodegenerative disorders, and renal failure (73). In Seonghun Kim’s study, GPX4 mRNA expression in kidney biopsy samples from diabetic patients was decreased compared to nondiabetic patients. Recent studies showed that tubular cell death and intrarenal interstitial edema and proteinuria were significantly increased in GPX4-deficient mice, suggesting that GPX4 has a renin protective effect in tubular cells (74). In the ferroptosis-induced RCD, hemochromatosis initiates lipid peroxidation under the circumstance with the inhibited activity of glutathione peroxidase-4 (GPX-4), which is responsible for plasma membrane disruption (73). Previous studies have noted that ferroptosis-related markers’ expression was increased, such as acyl-CoA synthase long-chain family member 4 (ACSL4), which is a potential biomarker and contributor to ferroptosis (73). It was recently reported by Wang et al. that a significant increase in ACSL4 expression and a decrease in Gpx4 expression were observed in DKD mice, including STZ-type and *db/db* mice (73). Lipid peroxidation products and iron content increased in the kidney of DKD mice (75). In addition, iron and high levels of ACSL4 sensitize ferroptosis, while ferroptosis inducers Erastin or RSL3 could induce renal tubular cell death *in vitro* (76). Similarly, another study found hemochromatosis decreased antioxidant capacity and produced large amounts of ROS and lipid peroxidation in STZ-induced DBA/2J diabetic mice and human proximal renal tubular (HK-2) cells cultured with high glucose, which are symbolic alternations in ferroptosis (77). In addition, they also observed the morphological changes of mitochondria characterized by iron death in cells cultured with high glucose (77). The addition of iron statin-1 (Fer-1) to the DKD model can significantly rescue the above alterations in diabetic mice and reduced pathological renal damage in diabetic mice (77). Therefore, ferroptosis could become a research direction to discover the potential treatments for DKD.

## Intersections Between Different Patterns of Regulated Cell Death

There is growing evidence to demonstrate that different RCD patterns are interconnected on multiple levels and should perhaps be viewed in less discrete terms. However, these different cell death patterns crosstalk each other, engaging with mitochondria. Autophagy and apoptosis, primarily through the role of Beclin-1, result in substantial crosstalk. The Beclin-1-Bcl2 complex generation is sufficient to suppress autophagy while interrupting the formation of the Beclin-1-Vps34 complex (78). Besides, in the process of apoptosis, caspases cleave Beclin-1 and inactivate autophagy (79), while its carboxyl-terminal fragment is relocated in mitochondria, which can thus promote the generation of cytochrome C (80). This offers an additional shutdown pathway to limit autophagy and an amplifying loop that promotes apoptosis. In addition to Beclin-1, other autophagy proteins can modulate the balance between autophagy and apoptosis. For example, Atg12 and Atg3 suppress apoptosis (81), while the cleavage of Atg5 produces pro-apoptotic fragments (82). Autophagy is generally considered a protective process. However, in some specific cases, it can damage tissue and induce cell death through apoptosis or necrosis (83). In contrast, excessive autophagy can also lead to apoptosis, which is harmful to the human body. The abnormal relationship between autophagy and apoptosis may cause the development of diabetic complications.

It has been reported that the accumulation of mitochondria and the aggregation of qstm1/p62 are improved in the damaged kidney of distal tubule specific autophagy-deficient mice, resulting in elevated levels of the NLRP3 inflammasome, IL-1β, and caspase-1. Moreover, autophagy is highly related to necroptosis, which can inhibit necroptosis directly by degrading RIPK1. In contrast, autophagy can be suppressed by RIPK1 through its effects on the TFEB transcription factor’s ERK phosphorylation (84). On the other hand, RIPK3 facilitates caspase8 to cleave p62, thus inhibiting LC3-p62 interaction in autophagy (85). About the relationship between ferroptosis and autophagy, ferroptosis has been recognized as a form of autophagy-dependent cell death in some conditions. for instance, ferritinophagy and lipophagy play a significant role in promoting ferroptosis (86). In addition, autophagy has been demonstrated to improve ferroptosis by degrading ferritin, resulting in a higher level of iron (87). On the contrary, their interaction also promotes survival through the additional function- of the p62-SQSTM1 autophagy receptor as an oxidative stress sensor (86).

In a recent study, Ferroptotic agent-induced PUMA (p53 upregulated modulator of apoptosis) plays an important role in the crosstalk between ferroptosis and apoptosis. In detail, ferroptotic agents induce ER stress and elevate the expression of the proapoptotic molecule PUMA through the ER stress-mediated PERK-eIF2α-ATF4-CHOP pathway without inducing apoptosis (88). however, Ferroptosis proceeds even in the absence of key effectors of apoptosis and necroptosis, such as BAX, BAK, caspases, MLKL, RIPK1, and RIPK3 (75).

Caspase-8 is essential for growth and progression, homeostasis, and prevention of tissue damage in adulthood, involved in the regulation and initiation of RCD mediated by the death receptor. Moreover, caspase-8 is a molecular switch controlling apoptosis, necroptosis, and pyroptosis. Necroptosis and apoptosis share the same upstream signaling pathway (89). RIPK1 is one of the upstream signal elements, which is considered to be the trigger switch of CASP8-dependent apoptosis or RIPK3/MLKL-dependent necroptosis (90). Under certain circumstances, RIPK3 can be used as a pro-apoptotic aptamer, utilizing the death domain (FADD) to recruit RIPK1 and Fas-related proteins to activate caspase-8, thereby leading to apoptosis. When RIPK3 is inactive or MLKL is missing, this effect depends on the participation of caspase-8 (91, 92). Both pyroptosis and necroptosis are hemolytic and inflammatory RCD that need to destroy the membrane and form membrane pores. The difference is that the pore formation of pyroptosis is mediated by GSDMD and necroptosis is mediated by MLKL. Both GSDMD and MLKL signals can result in potassium efflux, initiate NLRP3 inflammasome, and lead to IL-1β-mediated inflammatory response. It has been recently validated that RIPK3 is a substantial mediator for the generation of inflammatory bodies in NLRP3 (93). Some studies have suggested that RIPK3-induced NLRP3 inflammasome is correlated with necroptosis. In particular, RIPK3–MLKL initiates the activation of NLRP3 when caspase 8’s activity is inhibited (59). In general, RIPK3-mediated activation of NLRP3 inflammasome can be either necroptosis-dependent or independent, relying on the activity of caspase-8.

## Conclusion

DKD has a variety of origins and potential mechanisms. However, ACE inhibitors (ACEIs) or angiotensin II receptor blockers (ARBs) for anti-RAS therapy and blood glucose level control are applied as currently available treatments for DKD. Moreover, anti-RAS therapy only exhibits limited efficacy on DKD, partially due to renin’s upregulated expression levels. Due to the previous exposure to hyperglycemia recorded in metabolic memory, a single blood glucose control treatment cannot prevent kidney disease progression. Therefore, to slow down the progress of DKD, there is an unmet need to explore more effective treatments. In recent years, many studies have further demonstrated different RCD patterns in the renal tubular epithelial cells of DKD. Although significant progress has been achieved to better understand cell death’s underlying mechanisms in cellular and molecular levels, there is no research focused on treating the RCD signaling pathway except for the promising preclinical studies. Given the many different RCD patterns in DKD, it is necessary to consider combined therapy that blocks multiple regulatory pathways of cell death simultaneously or at specific time windows to guarantee cell survival and renal function.

## Author Contributions

SS and KW decided on the topics. SS and CJ reviewed the literature. SS wrote the manuscript. KW revised the manuscript. All authors read and approved the manuscript and met the criteria for authorship.

## Conflict of Interest

The authors declare that the research was conducted in the absence of any commercial or financial relationships that could be construed as a potential conflict of interest.

## Publisher’s Note

All claims expressed in this article are solely those of the authors and do not necessarily represent those of their affiliated organizations, or those of the publisher, the editors and the reviewers. Any product that may be evaluated in this article, or claim that may be made by its manufacturer, is not guaranteed or endorsed by the publisher.

## References

[1] Dos hi SM Friedman AN. Diagnosis and Management of Type 2 Diabetic Kidney Disease. Clin J Am Soc Nephrol (2017) 12(8):1366–73. doi: 10.2215/CJN.11111016 PMC5544517 28280116

[2] Epstein FH Remuzzi G Bertani T. Pathophysiology of Progressive Nephropathies. N Engl J Med (1998) 339(20):1448–56. doi: 10.1056/NEJM199811123392007 9811921

[3] Thomas MC Brownlee M Susztak K Sharma K Jandeleit-Dahm KA Zoungas S. Diabetic Kidney Disease. Nat Rev Dis Primers (2015) 1:15018. doi: 10.1038/nrdp.2015.18 27188921 PMC7724636

[4] Xiong Y Zhou L. The Signaling of Cellular Senescence in Diabetic Nephropathy. Oxid Med Cell Longev (2019) 2019:1–16. doi: 10.1155/2019/7495629 PMC6794967 31687085

[5] Tang D Kang R Berghe TV Vandenabeele P Kroemer G. The Molecular Machinery of Regulated Cell Death. Cell Res (2019) 29(5):347–64. doi: 10.1038/s41422-019-0164-5 PMC6796845 30948788

[6] Bedoui S Herold MJ Strasser A. Emerging Connectivity of Programmed Cell Death Pathways and its Physiological Implications. Nat Rev Mol Cell Biol (2020) 21:678–95. doi: 10.1038/s41580-020-0270-8 32873928

[7] Jennifer S Craig S Tara MM Ryan MP. New Developments Concerning the Proximal Tubule in Diabetic Nephropathy: *In Vitro* Models and Mechanisms. Nephrol Dialysis Transplant (2015) 30(4):60–7. doi: 10.1093/ndt/gfv264 26209740

[8] Mauer SM Steffes MW Ellis EN Sutherland DE Brown DM Goetz FC. Structural-Functional Relationships in Diabetic Nephropathy. J Clin Invest (1984) 74(4):1143–55. doi: 10.1172/JCI111523 PMC425280 6480821

[9] Chevalier RL. The Proximal Tubule is the Primary Target of Injury and Progression of Kidney Disease: Role of the Glomerulotubular Junction. Am J Physiol Renal Physiol (2016) 311(1):F145–61. doi: 10.1152/ajprenal.00164.2016 PMC4967168 27194714

[10] Vallon V. The Proximal Tubule in the Pathophysiology of the Diabetic Kidney. Am J Physiol Regul Integr Comp Physiol (2011) 300(5):R1009–22. doi: 10.1152/ajpregu.00809.2010 PMC3094037 21228342

[11] Gilbert RE. Proximal Tubulopathy: Prime Mover and Key Therapeutic Target in Diabetic Kidney Disease. Diabetes (2017) 66(4):791–800. doi: 10.2337/db16-0796 28325740

[12] Fu H Liu S Bastacky SI Wang X Tian XJ Zhou D. Diabetic Kidney Diseases Revisited: A New Perspective for a New Era. Mol Metab (2019) 30:250–63. doi: 10.1016/j.molmet.2019.10.005 PMC6838932 31767176

[13] Russo LM Sandoval RM Campos SB Molitoris BA Comper WD Brown D. Impaired Tubular Uptake Explains Albuminuria in Early Diabetic Nephropathy. J Am Soc Nephrol (2009) 20(3):489–94. doi: 10.1681/ASN.2008050503 PMC2653682 19118149

[14] White KE Marshall SM Bilous RW. Prevalence of Atubular Glomeruli in Type 2 Diabetic Patients With Nephropathy. Nephrol Dial Transplant (2008) 23(11):3539–45. doi: 10.1093/ndt/gfn351 18558622

[15] Hasegawa K Wakino S Simic P Sakamaki Y Minakuchi H Fujimura K. Renal Tubular Sirt1 Attenuates Diabetic Albuminuria by Epigenetically Suppressing Claudin-1 Overexpression in Podocytes. Nat Med (2013) 19(11):1496–504. doi: 10.1038/nm.3363 PMC4041199 24141423

[16] Nihalani D Susztak K. Sirt1-Claudin-1 Crosstalk Regulates Renal Function. Nat Med (2013) 19(11):1371–2. doi: 10.1038/nm.3386 24202384

[17] Kim SS Song SH Kim IJ Jeon YK Kim BH Kwak IS. Urinary Cystatin C and Tubular Proteinuria Predict Progression of Diabetic Nephropathy. Diabetes Care (2013) 36(3):656–61. doi: 10.2337/dc12-0849 PMC3579333 23093662

[18] Nunzia D'Onofrio Luigi Servillo Alfonso Giovane Rosario Casale Milena Vitiello Raffaele Marfella . Ergothioneine Oxidation in the Protection Against High-Glucose Induced Endothelial Senescence: Involvement of SIRT1 and SIRT6. Free Rad Biol Med (2016) 96:211–22. doi: 10.1016/j.freeradbiomed.2016.04.013 27101740

[19] KK A DN A HO B HH A TM C JY D. Hyperglycemia Causes Cellular Senescence *via* a SGLT2 and P21-Dependent Pathway in Proximal Tubules in the Early Stage of Diabetic Nephropathy. J Diabetes Complications (2014) 28(5):604–11. doi: 10.1016/j.jdiacomp.2014.05.010 PMC4153757 24996978

[20] Zhang X Chen X Wu D Liu W Wang J Feng Z. Downregulation of Connexin 43 Expression by High Glucose Induces Senescence in Glomerular Mesangial Cells. J Am Soc Nephrol (2006) 17(6):1532–42. doi: 10.1681/ASN.2005070776 16675599

[21] Liu J Yang JR Chen XM Cai GY Lin LR He YN. Impact of ER Stress-Regulated ATF4/p16 Signaling on the Premature Senescence of Renal Tubular Epithelial Cells in Diabetic Nephropathy. Am J Physiol Cell Physiol (2015) 308(8):621–30. doi: 10.1152/ajpcell.00096.2014 25567807

[22] Blackburn EH. Switching and Signaling at the Telomere. Cell (2001) 106(6):661–73. doi: 10.1016/S0092-8674(01)00492-5 11572773

[23] Sampson MJ Hughes DA. Chromosomal Telomere Attrition as a Mechanism for the Increased Risk of Epithelial Cancers and Senescent Phenotypes in Type 2 Diabetes. Diabetologia (2006) 49(8):1726–31. doi: 10.1007/s00125-006-0322-4 16791617

[24] Fyhrquist F Tiitu A Saijonmaa O Forsblom C Group OBOS. Telomere Length and Progression of Diabetic Nephropathy in Patients With Type 1 Diabetes. J Intern Med (2009) 267(3):278–86. doi: 10.1111/j.1365-2796.2009.02139.x 19563389

[25] Reichert S Stier A. Does Oxidative Stress Shorten Telomeres *In Vivo*? A Review. Biol Lett (2017) 13(12):20170463. doi: 10.1098/rsbl.2017.0463 29212750 PMC5746531

[26] Fouquerel E Lormand J Bose A Lee HT Kim GS Li J. Oxidative Guanine Base Damage Regulates Human Telomerase Activity. Nat Struct Mol Biol (2016) 23(12):1092–100. doi: 10.1038/nsmb.3319 PMC5140714 27820808

[27] Lans H Hoeijmakers JHJ. Genome Stability, Progressive Kidney Failure and Aging. Nat Genet (2012) 44(8):836–8. doi: 10.1038/ng.2363 22836089

[28] Berger SL Kouzarides T Shiekhattar R Shilatifard A. An Operational Definition of Epigenetics. Genes Dev (2009) 23(7):781–3. doi: 10.1101/gad.1787609 PMC3959995 19339683

[29] Keating ST Van Diepen JA Riksen NP El-Osta A. Epigenetics in Diabetic Nephropathy, Immunity and Metabolism. Diabetologia (2018) 61(1):6–20. doi: 10.1007/s00125-017-4490-1 29128937 PMC6448927

[30] Wanner N Bechtel-Walz W. Epigenetics of Kidney Disease. Cell Tissue Res (2017) 369(1):75–92. doi: 10.1007/s00441-017-2588-x 28286899

[31] Hayashi K Sasamura H Nakamura M Azegami T Itoh H. KLF4-Dependent Epigenetic Remodeling Modulates Podocyte Phenotypes and Attenuates Proteinuria. J Clin Invest (2014) 124(6):2523–37. doi: 10.1172/JCI69557 PMC4089466 24812666

[32] Sharma I Dutta RK Singh NK Kanwar YS. High Glucose-Induced Hypomethylation Promotes Binding of Sp-1 to Myo-Inositol Oxygenase. Am J Pathol (2017) 187(4):724–39. doi: 10.1016/j.ajpath.2016.12.011 PMC5397681 28208054

[33] Horvath S. DNA Methylation Age of Human Tissues and Cell Types. Genome Biol (2013) 14(10):115. doi: 10.1186/gb-2013-14-10-r115 24138928 PMC4015143

[34] Wauer T Simicek M Schubert A Komander D. Mechanism of Phospho-Ubiquitin-Induced PARKIN Activation. Nature (2015) 524(7565):370–4. doi: 10.1038/nature14879 PMC4984986 26161729

[35] Urakawa I Yamazaki Y Shimada T Iijima K Hasegawa H Okawa K. Klotho Converts Canonical FGF Receptor Into a Specific Receptor for FGF23. Nature (2006) 444(7120):770–4. doi: 10.1038/nature05315 17086194

[36] Ohnishi M Razzaque SM. Dietary and Genetic Evidence for Phosphate Toxicity Accelerating Mammalian Aging. FASEB J (2010) 24(9):3562–71. doi: 10.1096/fj.09-152488 PMC2923352 20418498

[37] Luo C Zhou S Zhou Z Liu Y Yang L Liu J. Wnt9a Promotes Renal Fibrosis by Accelerating Cellular Senescence in Tubular Epithelial Cells. J Am Soc Nephrol (2018) 29(4):1238–56. doi: 10.1681/ASN.2017050574 PMC5875944 29440280

[38] Wu J Guan TJ Zheng S Grosjean F Liu W Xiong H. Inhibition of Inflammation by Pentosan Polysulfate Impedes the Development and Progression of Severe Diabetic Nephropathy in Aging C57B6 Mice. Lab Invest (2011) 91(10):1459–71. doi: 10.1038/labinvest.2011.93 21808238

[39] Goldberg EL Dixit VD. Drivers of Age-Related Inflammation and Strategies for Healthspan Extension. Immunol Rev (2015) 265(1):63–74. doi: 10.1111/imr.12295 25879284 PMC4400872

[40] Zhan M Usman I Sun L Kanwar YS. Disruption of Renal Tubular Mitochondrial Quality Control by Myo-Inositol Oxygenase in Diabetic Kidney Disease. J Am Soc Nephrol (2015) 26(6):1304–21. doi: 10.1681/ASN.2014050457 PMC4446875 25270067

[41] Guo K Lu J Huang Y Wu M Jia W. Protective Role of PGC-1α in Diabetic Nephropathy Is Associated With the Inhibition of ROS Through Mitochondrial Dynamic Remodeling. PloS One (2015) 10(4):e0125176. doi: 10.1371/journal.pone.0125176 25853493 PMC4390193

[42] Riedl SJ Salvesen GS. The Apoptosome: Signalling Platform of Cell Death. Nat Rev Mol Cell Biol (2007) 8(5):405–13. doi: 10.1038/nrm2153 17377525

[43] Martindale JL Holbrook NJ. Cellular Response to Oxidative Stress: Signaling for Suicide and Survival. J Cell Physiol (2002) 192(1):1–15. doi: 10.1002/jcp.10119 12115731

[44] Tesch GH Ma FY Han Y Liles JT Breckenridge DG Nikolic-Paterson DJ. ASK1 Inhibitor Halts Progression of Diabetic Nephropathy in Nos3-Deficient Mice. Diabetes (2015) 64(11):3903–13. doi: 10.2337/db15-0384 26180085

[45] Kroemer G Levine B. Autophagic Cell Death: The Story of a Misnomer. Nat Rev Mol Cell Biol (2008) 9(12):1004–10. doi: 10.1038/nrm2529 PMC2727358 18971948

[46] Karch J Schips TG Maliken BD Brody MJ Sargent MA Kanisicak O. Autophagic Cell Death is Dependent on Lysosomal Membrane Permeability Through Bax and Bak. eLife (2017) 6:e30543. doi: 10.7554/eLife.30543 29148970 PMC5697932

[47] Levine B. Autophagy in Cell Death: An Innocent Convict? J Clin Invest (2005) 115(10):2679–88. doi: 10.1172/JCI26390 PMC1236698 16200202

[48] Mizushima N Yoshimori T Levine B. Methods in Mammalian Autophagy Research. Cell (2010) 140(3):313–26. doi: 10.1016/j.cell.2010.01.028 PMC2852113 20144757

[49] Moto K Satohiro M. Role of mTOR Inhibitors in Kidney Disease. Int J Mol Sci (2016) 17(6):975. doi: 10.3390/ijms17060975 PMC4926507 27338360

[50] Preiss D Sattar N. Research Digest: SGLT2 Inhibition in Kidney and Liver Disease-Science Direct. Lancet Diabetes Endocrinol (2019) 7(6):427. doi: 10.1016/S2213-8587(19)30160-3 31103176

[51] Yu MW Leventhal JS Cravedi P. Totally Tubular, Dude: Rethinking DKD Pathogenesis in the Wake of SGLT2i Data. J Nephrol (2021) 34(3):629–31. doi: 10.1007/s40620-020-00868-0 32965657

[52] Cassis P Locatelli M Cerullo D Corna D Buelli S Zanchi C. SGLT2 Inhibitor Dapagliflozin Limits Podocyte Damage in Proteinuric non-Diabetic Nephropathy. JCI Insight (2018) 3(15):e98720. doi: 10.1172/jci.insight.98720 PMC6129124 30089717

[53] Yang C Chen XC Li ZH Wu HL Liu HF. SMAD3 Promotes Autophagy Dysregulation by Triggering Lysosome Depletion in Tubular Epithelial Cells in Diabetic Nephropathy. Autophagy (2020) 17(9):2325–44. doi: 10.1080/15548627.2020.1824694 PMC8496726 33043774

[54] Xu Y Liu L Xin W Zhao X Chen L Zhen J. The Renoprotective Role of Autophagy Activation in Proximal Tubular Epithelial Cells in Diabetic Nephropathy. J Diabetes Complications (2015) 29(8):976–83. doi: 10.1016/j.jdiacomp.2015.07.021 26297217

[55] Chan KM Luz NF Moriwaki K. Programmed Necrosis in the Cross Talk of Cell Death and Inflammation. Annu Rev Immunol (2015) 33(1):79–106. doi: 10.1146/annurev-immunol-032414-112248 25493335 PMC4394030

[56] Moriwaki K Chan KM. Necroptosis-Independent Signaling by the RIP Kinases in Inflammation. Cell Mol Life Sci (2016) 73(11-12):2325–34. doi: 10.1007/s00018-016-2203-4 PMC4889460 27048814

[57] Galluzzi L Kepp O Trojel-Hansen C Kroemer G. Non-Apoptotic Functions of Apoptosis-Regulatory Proteins. EMBO Rep (2012) 13(4):322–30. doi: 10.1038/embor.2012.19 PMC3321152 22402666

[58] Moreno-Gonzalez G Vandenabeele P Krysko DV. Necroptosis: A Novel Cell Death Modality and its Potential Relevance for Critical Care Medicine. Am J Respir Crit Care Med (2016) 194(4):415–28. doi: 10.1164/rccm.201510-2106CI 27285640

[59] Lawlor KE Khan N Mildenhall A Gerlic M Croker BA D’Cruz AA. RIPK3 Promotes Cell Death and NLRP3 Inflammasome Activation in the Absence of MLKL. Nat Commun (2015) 6(1):6282. doi: 10.1038/ncomms7282 25693118 PMC4346630

[60] Zhu Y Cui H Lv J Li G Zhong L. Angiotensin II Triggers RIPK3-MLKL-Mediated Necroptosis by Activating the Fas/FasL Signaling Pathway in Renal Tubular Cells. PloS One (2020) 15(3):e0228385. doi: 10.1371/journal.pone.0228385 32134954 PMC7058379

[61] Sharma R Sharma M Reddy S Savin VJ Nagaria AM Wiegmann TB. Chronically Increased Intrarenal Angiotensin II Causes Nephropathy in an Animal Model of Type 2 Diabetes. Front Biosci (2006) 11(1):968–76. doi: 10.2741/1853 16146787

[62] Lovshin JA Boulet G Lytvyn Y Lovblom LE Bjornstad P Farooqi MA. Renin-Angiotensin-Aldosterone System Activation in Long-Standing Type 1 Diabetes. JCI Insight (2018) 3(1):e96968. doi: 10.1172/jci.insight.96968 PMC5821172 29321380

[63] Giuseppina B Marie SA Raffaele DC. Advanced Glycation End Products and Vascular Inflammation: Implications for Accelerated Atherosclerosis in Diabetes. Cardiovasc Res (2004) 63(4):582–92. doi: 10.1016/j.cardiores.2004.05.001 15306213

[64] Sassy-Prigent C Heudes D Mandet C Belair MF Michel O Perdereau B. Early Glomerular Macrophage Recruitment in Streptozotocin-Induced Diabetic Rats. Diabetes (2000) 49(3):466–75. doi: 10.2337/diabetes.49.3.466 10868970

[65] Bauernfeind F Bartok E Rieger A Franchi L Nunez G Hornung V. Cutting Edge: Reactive Oxygen Species Inhibitors Block Priming, But Not Activation, of the NLRP3 Inflammasome. J Immunol (2011) 187(2):613–7. doi: 10.4049/jimmunol.1100613 PMC3131480 21677136

[66] Wang J Wen Y Lv LL Liu H Tang RN Ma KL. Involvement of Endoplasmic Reticulum Stress in Angiotensin II-Induced NLRP3 Inflammasome Activation in Human Renal Proximal Tubular Cells. vitro. Acta Pharmacol Sin (2015) 36(7):821–30. doi: 10.1038/aps.2015.21 PMC4813084 26005910

[67] Wang W Wang X Chun J Vilaysane A Clark S French G. Inflammasome-Independent NLRP3 Augments TGF-β Signaling in Kidney Epithelium. J Immunol (2013) 190(3):1239–49. doi: 10.4049/jimmunol.1201959 23264657

[68] Wang C Pan Y Zhang QY Wang FM Kong LD. Quercetin and Allopurinol Ameliorate Kidney Injury in STZ-Treated Rats With Regulation of Renal NLRP3 Inflammasome Activation and Lipid Accumulation. PloS One (2012) 7(6):e38285. doi: 10.1371/journal.pone.0038285 22701621 PMC3372527

[69] Zhou R Yazdi AS Menu P Tschopp J. A Role for Mitochondria in NLRP3 Inflammasome Activation. Nature (2011) 469(7329):221–5. doi: 10.1038/nature09663 21124315

[70] Kose T Vera-Aviles M Sharp PA Latunde Dada GO. Curcumin and (-)- Epigallocatechin-3-Gallate Protect Murine MIN6 Pancreatic Beta-Cells Against Iron Toxicity and Erastin-Induced Ferroptosis. Pharmaceuticals (2019) 12(1):26. doi: 10.3390/ph12010026 PMC6469157 30736288

[71] Basuli D Stevens RG Torti FM Torti SV. Epidemiological Associations Between Iron and Cardiovascular Disease and Diabetes. Front Pharmacol (2014) 5:117. doi: 10.3389/fphar.2014.00117 24904420 PMC4033158

[72] Stoyanovsky DA Tyurina YY Shrivastava I Bahar I Tyurin VA Protchenko O. Iron Catalysis of Lipid Peroxidation in Ferroptosis: Regulated Enzymatic or Random Free Radical Reaction? Free Radic Biol Med (2019) 133:153–614. doi: 10.1016/j.freeradbiomed.2018.09.008 30217775 PMC6555767

[73] Wang Y Bi R Quan F Cao Q Zhang D. Ferroptosis Involves in Renal Tubular Cell Death in Diabetic Nephropathy. Eur J Pharmacol (2020) 888:173574. doi: 10.1016/j.ejphar.2020.173574 32976829

[74] Kim S Kang SW Joo J Han SH Shin H Nam BY. Characterization of Ferroptosis in Kidney Tubular Cell Death Under Diabetic Conditions. Cell Death Dis (2021) 12(2):160. doi: 10.1038/s41419-021-03452-x 33558472 PMC7870666

[75] Stockwell BR Angeli JPF Bayir H. Ferroptosis: A Regulated Cell Death Nexus Linking Metabolism, Redox Biology, and Disease. Cell (2017) 171(2):273–85. doi: 10.1016/j.cell.2017.09.021 PMC5685180 28985560

[76] Feng X Wang S Sun Z Dong H Yu H Huang M. Ferroptosis Enhanced Diabetic Renal Tubular Injury *via* HIF-1α/HO-1 Pathway in Db/Db Mice. Front Endocrinol (2021) 12:626390. doi: 10.3389/fendo.2021.626390 PMC7930496 33679620

[77] Li S Zheng L Zhang J Liu X Wu Z. Inhibition of Ferroptosis by Up-Regulating Nrf2 Delayed the Progression of Diabetic Nephropathy. Free Radic Biol Med (2021) 162:435–49. doi: 10.1016/j.freeradbiomed.2020.10.323 33152439

[78] Maiuri M. Functional and Physical Interaction Between Bcl-X (L) and BH3-Like Domain in Beclin-1. EMBO J (2007) 26:2527–39. doi: 10.1038/sj.emboj.7601689 PMC1868901 17446862

[79] Luo S Rubinsztein DC. Apoptosis Blocks Beclin 1-Dependent Autophagosome Synthesis: An Effect Rescued by Bcl-Xl. Cell Death Differ (2010) 17(2):268–77. doi: 10.1038/cdd.2009.121 PMC2894406 19713971

[80] Wirawan E Vande WL Kersse K Cornelis S Claerhout S Vanoverberghe I. Caspase-Mediated Cleavage of Beclin-1 Inactivates Beclin-1-Induced Autophagy and Enhances Apoptosis by Promoting the Release of Proapoptotic Factors From Mitochondria. Cell Death Dis (2010) 1(1):e18. doi: 10.1038/cddis.2009.16 21364619 PMC3032505

[81] Radoshevich L Murrow L Chen N Fernandez E Roy S Fung C. ATG12 Conjugation to ATG3 Regulates Mitochondrial Homeostasis and Cell Death. Cell (2010) 142(4):590–600. doi: 10.1016/j.cell.2010.07.018 20723759 PMC2925044

[82] Yousefi S Perozzo R Schmid I Ziemiecki A Schaffner T Scapozza L. Calpain-Mediated Cleavage of Atg5 Switches Autophagy to Apoptosis. Nat Cell Biol (2006) 8(10):1124–32. doi: 10.1038/ncb1482 16998475

[83] Kevin B Robin M Alexandria L Kamphorst JJ Jing F Jim C. Autophagy Suppresses RIP Kinase-Dependent Necrosis Enabling Survival to mTOR Inhibition. PloS One (2012) 7(7):e41831. doi: 10.1371/journal.pone.0041831 22848625 PMC3406086

[84] Yonekawa T Gamez G Kim J Tan AC Thorburn J Gump J. RIP1 Negatively Regulates Basal Autophagic Flux Through TFEB to Control Sensitivity to Apoptosis. EMBO Rep (2015) 16(6):700–8. doi: 10.15252/embr.201439496 PMC4467854 25908842

[85] Matsuzawa Y Oshima S Nibe Y Kobayashi M Maeyashiki C Nemoto Y. RIPK3 Regulates P62-LC3 Complex Formation *via* the Caspase-8-Dependent Cleavage of P62. Biochem Biophys Res Commun (2015) 456(1):298–304. doi: 10.1016/j.bbrc.2014.11.075 25450619

[86] Liu J Kuang F Kroemer G Klionsky D Kang R Tang D. Autophagy-Dependent Ferroptosis: Machinery and Regulation. Cell Chem Biol (2020) 27(4):420–35. doi: 10.1016/j.chembiol.2020.02.005 PMC7166192 32160513

[87] Hou W Xie Y Song X Sun X Lotze M Zeh H. Autophagy Promotes Ferroptosis by Degradation of Ferritin. Autophagy (2016) 12(8):1425–8. doi: 10.1080/15548627.2016.1187366 PMC4968231 27245739

[88] Ghosh AP Klocke BJ Ballestas ME Roth KA. CHOP Potentially Co-Operates With FOXO3a in Neuronal Cells to Regulate PUMA and BIM Expression in Response to ER Stress. PloS One (2012) 7:e39586. doi: 10.1371/journal.pone.0039586 22761832 PMC3386252

[89] Takahashi N Vereecke L Bertrand MJM Duprez L Berger SB Divert T. RIPK1 Ensures Intestinal Homeostasis by Protecting the Epithelium Against Apoptosis. Nature (2014) 513(7516):95–9. doi: 10.1038/nature13706 25186904

[90] Zhang DW Shao J Lin J Zhang N Lu BJ Lin SC. RIP3, an Energy Metabolism Regulator That Switches TNF-Induced Cell Death From Apoptosis to Necrosis. Science (2009) 325(5938):332–6. doi: 10.1126/science.1172308 19498109

[91] Newton K Dugger DL Wickliffe KE Kapoor N Almagro MCD Vucic D. Activity of Protein Kinase RIPK3 Determines Whether Cells Die by Necroptosis or Apoptosis. Science (2014) 343(6177):1357–60. doi: 10.1126/science.1249361 24557836

[92] Mandal P. RIP3 Induces Apoptosis Independent of Pronecrotic Kinase Activity. Mol Cell (2014) 56(4):481–95. doi: 10.1016/j.molcel.2014.10.021 PMC4512186 25459880

[93] Wang X Jiang W Yan Y Gong T Han J Tian Z. RNA Viruses Promote Activation of the NLRP3 Inflammasome Through a RIP1-RIP3-DRP1 Signaling Pathway. Nat Immunol (2014) 15(12):1126–33. doi: 10.1038/ni.3015 25326752

